# Simoultaneous bilateral medial opening wedge high tibial osteotomy can be performed safely and effectively without bone grafting: analysis of a monocentric retrospective series

**DOI:** 10.1186/s12891-024-08022-8

**Published:** 2024-12-19

**Authors:** Daniele Screpis, Marco Baldini, Gianluca Piovan, Fabio Santamaria, Venanzio Iacono, Antonio Gigante, Claudio Zorzi

**Affiliations:** 1 IRCCS Ospedale Sacro Cuore—Don Calabria; Viale Luigi Rizzardi 4, Negrar, VR Italia; 2 Clinica Ortopedica dell’adulto e pediatrica, Università Politecnica delle Marche, Via Tronto 10/A, Ancona, AN Italia; 3 AST Pesaro UrbinoOspedale San Salvatore Pesaro, Piazzale Cinelli 4, Pesaro, PU, Marche Italy

**Keywords:** High tibial osteotomy, Bone healing, Tibial varus, Medial knee osteoarthritis

## Abstract

**Background:**

Simultaneous bilateral high tibial osteotomy (SBHTO) is a potential solution to treat bilateral medial osteoarthritis (OA) associated with tibial varus deformity. Concerns on the potential problems related to bone healing exists, and most of the surgeon performing SBHTO use bone void filler as associated procedure.

This paper aim to evaluate safety and efficacy of SBHTO using locking plate, autologous cancellous bone mobilization and no bone void filler with immediate weight bearing at tolerance protocol.

**Methods:**

Consecutive patients performing primary SBHTO between January 2019 and December 2022 were retrospectively evaluated. Functional and pain score, subjective satisfaction and complications were noted at 2, 3, 6 months and final follow up, with a minimum of 12 months.

**Results:**

A total of 40 patients (80 knees) were included. Mean correction for each limb was 8.67° ± 2.24°. No patients presented with major complications. Pain was reduced but activity level worsened 2 months after surgery. All pain and functional scores improved significantly from months 3 up to final follow up. 95% of patients reported to be subjectively satisfied with surgery.

**Conclusions:**

This paper showed that SBHTO can be performed safely and with good results without bone grafting the osteotomy gap, even for correction up to 12°. Pain improved already 2 months after surgery, while activity level and function start to improve at 3 months after surgery.

**Level of evidence:**

IV.

## Background

High tibial osteotomy (HTO) is a valid procedure to treat medial symptomatic knee osteoarthritis (OA) associated with extrarticular varus malalignement.

With the introduction of locking plates, providing more stable fixation, medial opening wedge high tibial osteotomy (OWHTO) technique has gained popularity because is less technically demanding and allows a fine regulation of the correction, without risks for peroneal nerve injury [1].

Since varus is mostly a constitutional deformity, many patients present with bilateral medial knee OA [2].

In this setting, the most common practice is to do a staged procedure, performing surgery on the contralateral side in a second time, even though a precise timing for the second step is not clear.

Staged procedure, though, may leave the patient with a painful contralateral side, a subtle leg length discrepancy and alignment differences between legs, that may impair rehabilitation [3, 4]. Moreover, a second anesthesia, surgical act and a new rehabilitation is waiting the patient right at the end of the first recovery.

Simultaneous bilateral medial opening wedge osteotomy (SBHTO) has been described as a potential solution to accelerate recovery and avoid a second surgery in patient with bilateral symptomatic medial OA, similarly to the concept of simultaneous bilateral total knee arthroplasty (TKA) [5, 6].

Many surgeons still use bone grafting in their HTO routine with a consistent increase of costs and potential harm to the patient. In fact, autologous bone grafting is related to donor site morbidity, and synthetic grafts are expensive and often associated with infection, bone collapse and nonunion [7–9],

While in the setting of monolateral HTO the evidence of unusefulness of bone grafting is consistent, in the setting of SBHTO the need for bone grafting seems to be more debatable, given the absence of a “healthy” contralateral side that can support bodyweight during rehabilitation. In fact, among the few published studies on the use of SBHTO, none of the author reported to perform this surgery without bone grafting the osteotomy gap [10–14].

Our department has a high volume of HTO and nearly all of the OW procedures are performed with a cancellous bone mobilization technique and no bone grafting, with excellent results [15]. The proof of safety and efficacy in performing also SBHTO without bone grafting could be of interest for knee surgeon.

Our hypothesis is that SBHTO can be performed safely and with good results without bone grafting the osteotomy gap. Thus, we present our series of consecutive cases of SBHTO with a focus on early outcomes and complications.

## Materials and methods

Database of the “Sacro cuore—Don Calabria” Hospital of Negrar was mined searching for patients who underwent a primary SBHTO between January 2019 and December 2022. All patients gave informed consent to the treatment of their anonymised personal data for research purpose. Treatment of anonymised personal data for the purpose of this research was approved by local IRB.

A retrospective analysis of prospectively collected data was performed, according to the sequent inclusion and exclusion criteria.

Inclusion criteria were: (1) isolated bilateral medial compartment OA with a Kellgren Lawrence grade II or III [16], (2) an extraarticular varus deformity confirmed by an Hip-Knee-Ankle angle (HKAa) of > 5°, associated with a medial proximal tibial angle (MPTA) of < 85°, (3) patients complaining about bilateral symptoms; (4) availability of preoperative Tegner activity scale (TAS), Lysholm knee score (LKS) and Numeric Rating Scale (NRS) and (5) at least 1-year follow up. Asymptomatic mild patellofemoral and lateral degeneration were not considered a contraindication to the procedure. Patients with lateral compartment subluxation were excluded.

If concomitant relevant femoral deformity was present, and planned tibial correction would have led to an MPTA of > 95°, a double level osteotomy (DLO) was considered, and the patients was excluded from the study. All these indications were considered in line with the more recently published in the ESSKA consensus on osteotomy around the painful knee [17].

Exclusion criteria included: (1) concomitant ligament injury, (2) concomitant cartilage reconstructive surgery, concomitant meniscal repair or meniscal allograft transplantation, (3) concomitant surgery or conditions interfering with postoperative rehabilitation program, (4), absence of preoperative or postoperative information. Presence of a relevant range of motion restriction (ROM) (i.e. fixed flexion deformity of > 5° or flexion < 110°), body mass index (BMI) > 35, diabetes mellitus (DM), known or highly suspected reumathic desease were considered an exclusion criteria for performing an OWHTO. Smoking was not considered a contraindication to the procedure, but the patients was prescribed to quit smoking for at least 6 months preoperative and during the entire postoperative year.

All patients fulfilling inclusion criteria were proposed to perform a SBHTO procedure. Patients willing to undergo a monolateral staged procedure were excluded from data recording. All consecutive patients undergoing SBHTO in the analysed period were included in the retrospective evaluation.

### Data collection and outcomes

Patient’s age, sex, BMI, preoperative TAS, LKS and NRS are collected as routine [18]. All patients gave informed consent to the use of their anonymized clinical data for the purpose of this research, according to the declaration of Helsinki.

Kellgren—Lawrence (KL) was used to assess OA severity on preoperative weight bearing x-Ray. Hip-Knee-Ankle angle (HKA) was calculated on preoperative long-standing radiographs. All patients performed MRI prior to surgery, to assess for concomitant associated lesions and to evaluate status of the lateral and patellofemoral compartment. Clinical examination to rule out instability was performed in every patient.

**Fig. 1:**
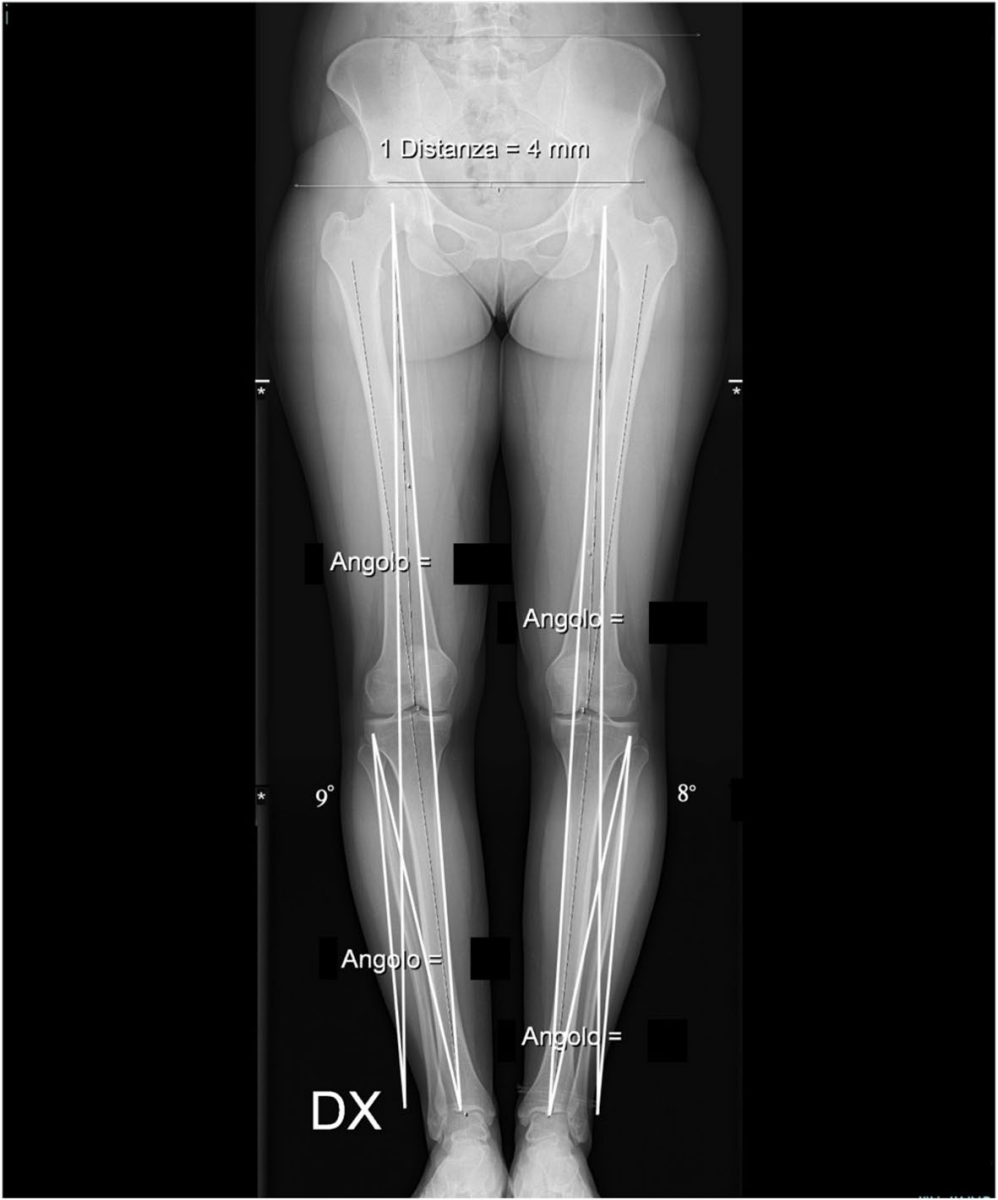
Preoperative planning for bilateral HTO: preoperative planning for bilateral medial opening wedge high tibial osteotomy using the Miniaci’s method

Preoperative planning of the osteotomy was performed using the Miniaci’s method [19]. (Fig. 1).

Stability correction evaluation was performed measuring the degree of the “open wedge” osteotomy angle on AP Xray, utilising the same “triangle method” proposed by Brosset et al. [20]. Measurements were performed immediately after surgery and then repeated postoperatively at 2 months and at 6 months and variation of more than 1° was noted.

Activity level, function and pain were evaluated after 2, 3, 6 months and at last follow up, after a minimum of 1 year, using, respectively, TAS, LKS and NRS. Patient reported satisfaction was evaluated at the end of follow up, asking a yes/no question: “*Have this surgery significantly improved your quality of life?*”.

**Fig. 2:**
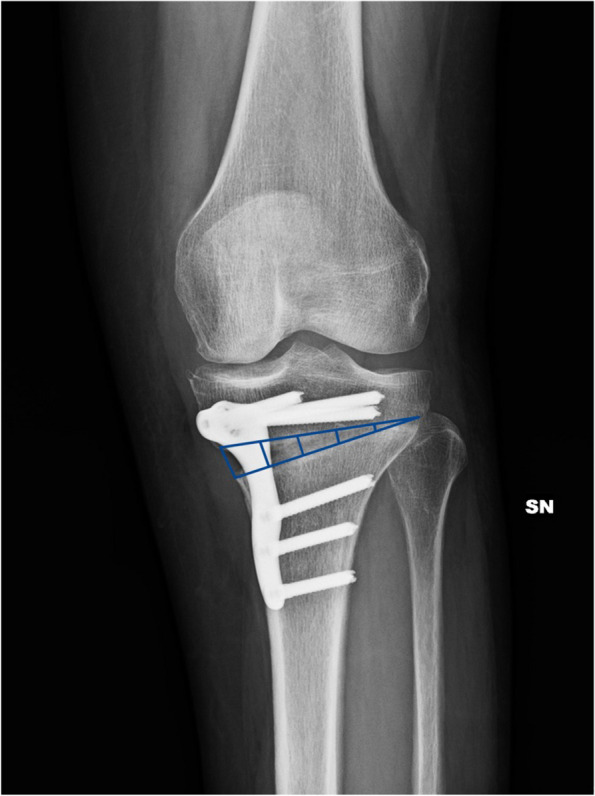
Evaluation of gap healing: Evaluation of bone ingrowth in osteotomy gap according to Brosset et al. The gap is divided in 5 sections of equal length. Bone ingrowth progresses from lateral to medial. The osteotomy is considered stable when the callus has reached zone 3, with more than 40% of the gap healed. In this figure, at 3 months follow up, bone callus is in zone 4, with nearly 80% of the gap healed

Evaluation of bone union was performed using radiological assessment as previously described by Brosset et al. [20] Briefly, using digitalized anteroposterior (AP) Rx, osteotomy space is represented with a triangle with the sides being the border of the osteotomy wedge. From lateral to medial, the triangle is divided into 5 areas of equal width. Osteotomy is considered stable with bone callus reaching zone 3 (40 – 60% of the gap healed), and complete union with bone callus reaching zone 5 (Fig. 2).

Stability was investigated at 2, 3, 6 months and 12 months and expressed as proportion of patients reaching, respectively, stability (zone 3) and complete union (zone 5).

Complications were noted, including infection, deep vein thrombosis (DVT), persistent pain and delayed union or nonunion. Minor self-reported complications were noted.

Lateral hinge fractures were assessed either intraoperatively or postoperatively and classified according to Takeuchi et al. [21] As routine in our centre, hinge fractures type I and II follow the same postoperative protocol whereas type III fractures are managed with protected weight-bearing (WB) for 6 weeks and progressive WB allowed after clinical and radiological evaluation.

### Surgical technique

All the procedures were performed by the same surgeons in a high volume centre with more than 150 HTO per year.

A diagnostic arthroscopy was routinary performed to assess cartilage degeneration in all three compartment and evaluating associated intraarticular lesions.

A monoplanar, medial opening—wedge HTO was performed in all patients, using the same angular plate for stabilization (*NCT- NewClip Technics, 44115 Haute-Goulaine, France*). Type 1 (6 holes) or Type 2 (8 holes) plate was chosen based on patients’ weight, according to manufacturer indications. At the end of the procedure, in all patients a cancellous bone mobilization procedure was performed. Briefly, with a narrow scalpel the cancellous bone from metaphysis and from part of the epiphysis is elevated and mobilized to partially fill the osteotomy gap, acting as a local autograft. In none of the patients a bone graft was used to fill the gap.

Tranexamic Acid (TXA) 1 g iv was administered immediately prior to surgery. A small drain was used in all patients and removed 24-48 h post surgery. Standard anti deep venous thrombosis (DVT) prophylaxis was performed with low molecular weight heparin (LMWH) 4000UI/die subcutaneously.

During the postoperative period, knee brace was prescript for 4 weeks. During the first 2 weeks range of motion (ROM) was restricted to 0–90°. After 2 weeks, patients were allowed complete ROM, starting a specific rehabilitation program. FWB as tolerated was permitted from the day after surgery, without any limitation on the use of crutches. Limitation on high impact activity such as running and jumping was prescript for at least 2 months post surgery.

### Statistical analysis

Continuous numeric data are reported as mean and standard deviation.

Group comparison for LKS, TAS and NRS and other continuous data are performed using T-test for dependent groups. Statistical significance is set at *p* < 0.05.

A priori power analysis was performed using G*Power. To obtain a study power of 0.95, with an alpha error of 0.05 and an effect size of 0.6, using a T test for matched pairs, a total sample size of 32 patients was required.

## Results

A total of 40 patients were included in the retrospective analysis (80 knees), with a mean follow up of 23.4 ± 9.9 months. Age, BMI, HKA, KL and preoperative functional scores are reported in Table 1.

**Table 1:** Preoperative clinical data

	MEAN ± SD	RANGE
Age	45.6 ± 14.92 (years)	20 – 66
BMI	26.15 ± 4.89	19 – 36
KL dx	2,55 ± 0,50	2 – 3
KL sn	2,42 ± 0,51	2 – 3
TAS score	2.45 ± 0.67	2 – 4
Lysholm	53.7 ± 9.33	34 – 66
NRS	7.5 ± 1.09	5 – 9
HKA	171° ± 2.1°	168° – 176°
Follow Up	23.4 ± 9.96 (months)	12 – 42 (months)

Preoperative clinical and demographical data, reported as mean and standard deviation (SD) and range

*BMI* Body mass index, *TAS* Tegner activity scale, *NRS* Numeric rating scale, *HKA* Hip-Knee-Ankle angle

**Fig. 3:**
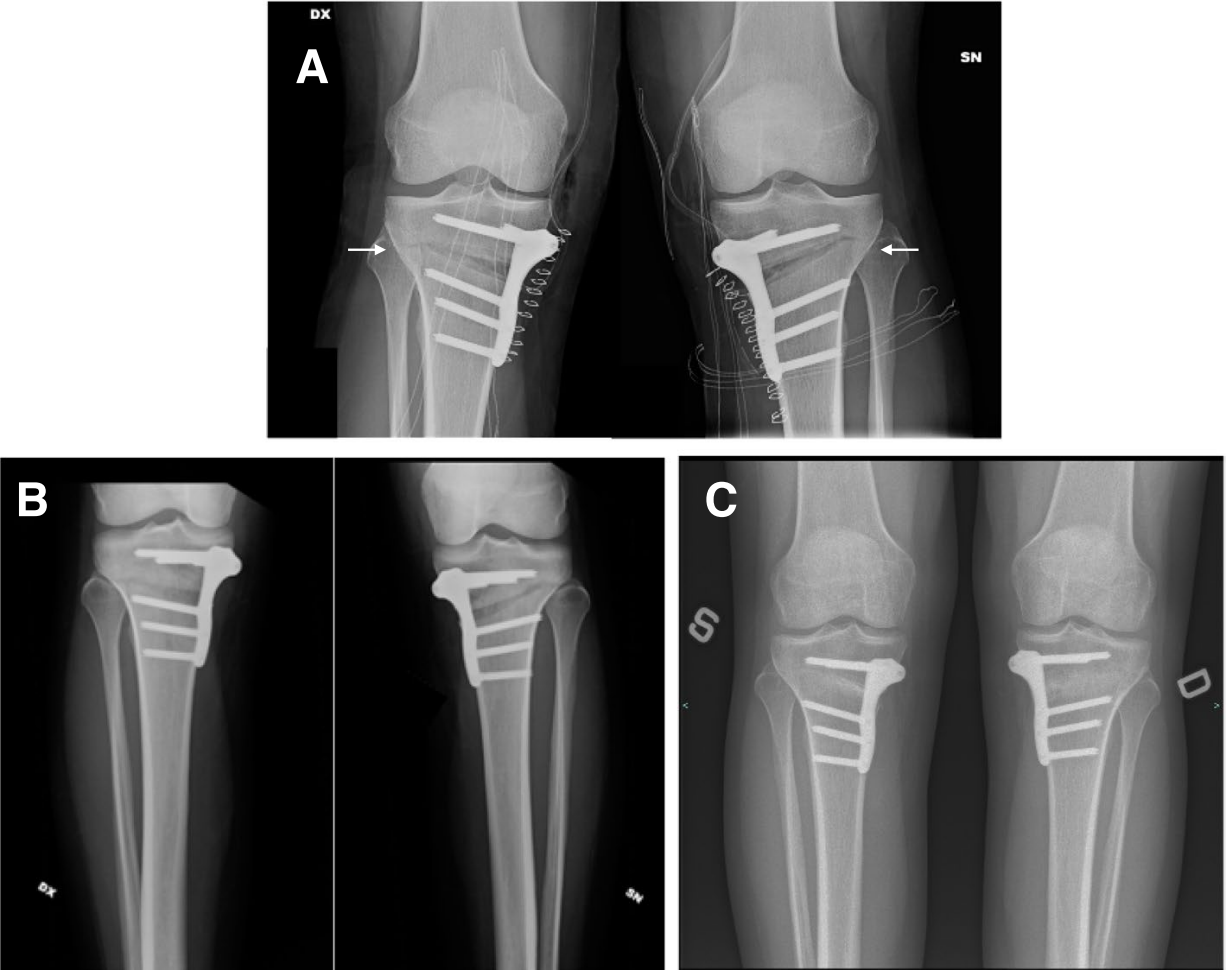
Radiological progression of gap healing. **A** Postoperative x-Ray of SBHTO showing a type 2 Takeuchi hinge fracture. (white arrows). **B** X-rays at 2 months postoperative. No displacement. Bone callus is evident up to zone 4 bilaterally. **C** X-ray at around 6 months after surgery shows complete bone healing bilaterally

In all patients, regardless of hinge fracture, stability was reached at 3 months, and complete bone healing occurred within 6 months without complications, according to the evaluation method proposed by Brosset et al. (Fig. 3 A, B, C).

TAS demonstrated a significant reduction at 2 months postoperative, with patients showing at the same time a significant improvement in pain score and a slight increase in LKS, that did not reach statistical significance.

**Fig. 4:**
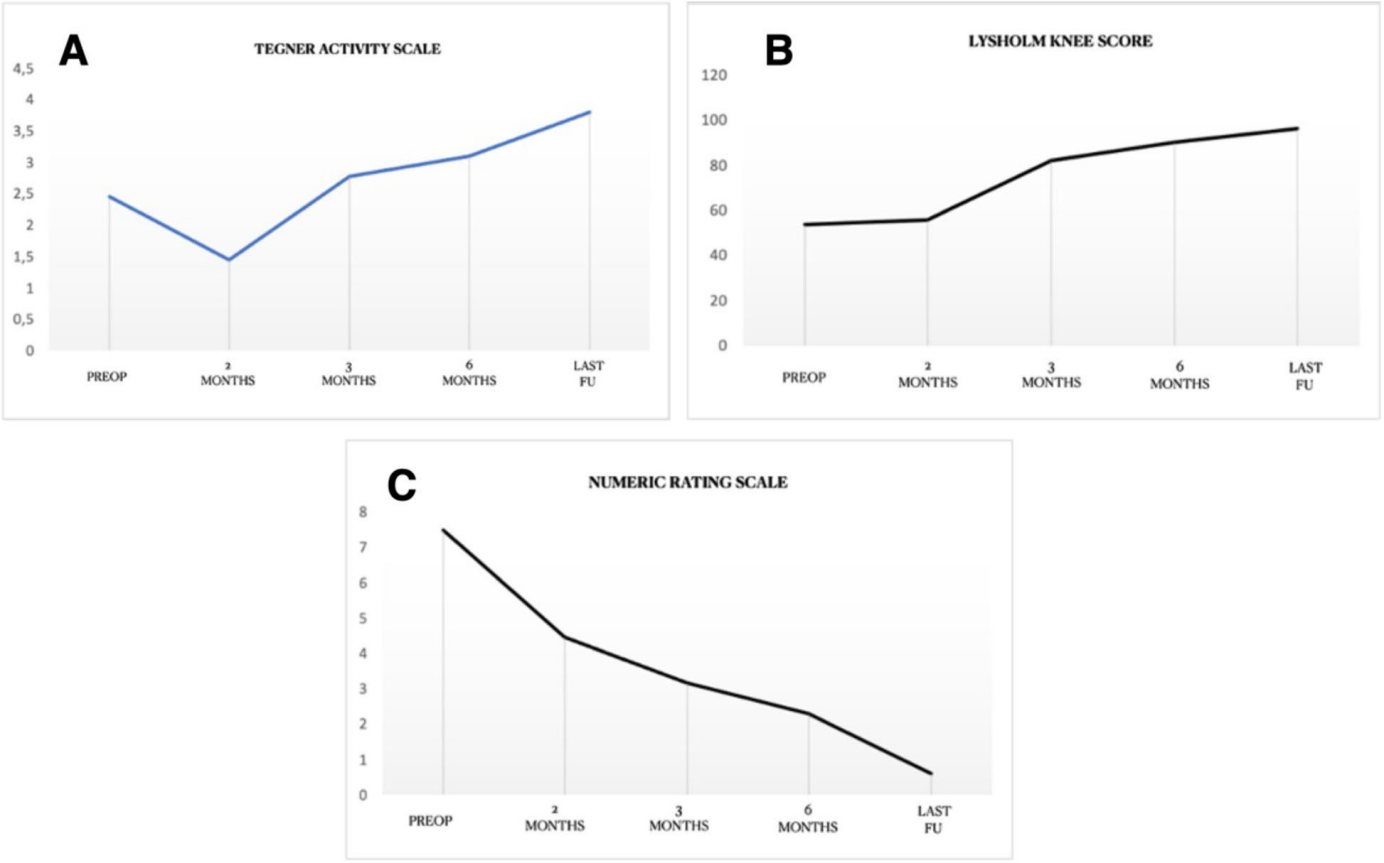
**P**ain and functional scores progression at follow up. **A** Progression of Tegner activity scale during follow up. **B** Progression of Lysholm knee score during follow up. **C** Progression of Numeric rating scale for pain during follow up

At 3 and 6 months all scores demonstrated a significant improvement as compared to baseline, with further improvement up to final follow up (Fig. 4 A, B, C).

TAS, LKS and NRS during follow up are reported in Table 2.

**Table 2:** Clinical and functional scores reported during follow up

	TAS		LKS		NRS	
	Mean ± SD	*p*	Mean ± SD	*p*	Mean ± SD	*p*
PREOP	2.45 ± 0.67	-	53.7 ± 9.33	-	7.5 ± 1.09	-
2 MONTHS	1.45 ± 0.50	*< 0.05*	55.9 ± 7.03	*ns*	4.45 ± 0.67	*ns*
3 MONTHS	2.77 ± 0.62	*<0.001*	82.1 ± 8.23	*<0.001*	3.18 ± 0.71	*<0.001*
6 MONTHS	3.1 ± 0.59	*<0.001*	90.37 ± 10.24	*<0.001*	2.3 ± 0.91	*<0.001*
LAST FU	3.8 ± 1.26	*<0.001*	96.4 ± 11.18	*<0.001*	0.6 ± 1.08	*<0.001*

Patients’ reported outcomes prior to surgery and during follow up at 2, 3, 6 months and last follow up (minimum 12 months). Statistical significance is set at *p* < 0.05

*TAS* Tegner Activity Scale, *LKS* Lysholm Knee Score; Numeric Rating Scale, *SD* Standard deviation, *FU* Follow up

Mean correction for each limb was 9.15° ± 2.34° for the right side and 8.20° ± 2.07° for the left side. At 2 months postoperatively, osteotomy angle was respectively 9.1° ± 2.23° and 8.15° ± 1.93° for the right and left side.

Magnitude of correction was stable in all the patients, and no significant changes of osteotomy angle was reported during follow up.

At final follow up, an overall 95% rate of “subjective satisfaction” was reported, with only two patients answering “NO” to the final yes/noquestion.

No major complications were reported, including DVT, nonunion or infections. 15 patients reported dysesthesia around the surgical incision persisting at 6 months, and 21 patients referred during follow up complaining about discomfort related to implant that resolved after plate removal.

Lateral hinge fracture (LHF) occured in 22 cases. Of those, 16 were classified as type I and 6 as type II. No type III fracture was reported; thus, all the patients underwent the same postoperative protocol.

## Discussion

To the best of our knowledge this is the first study to report on a series of simultaneous bilateral opening wedge high tibial osteotomy performed without using any type of bone grafting to fill the osteotomy gap.

The main findings of this paper are the proof of feasibility, safety and good results obtained in the setting of SB-OWHTO performed without any type of bone void filler.

Performing an HTO without graft may prevent donor site morbidity, when an autologous graft is adopted, or, on the other hand, may reduce the risk of nonunion or infection, if a synthetic graft is used [9]. Potentially, there may also be a reduction in costs and a faster surgical procedure, even if this is still not demonstrated.

While the number of surgeons performing medial OWHTO is growing, there is still no unified indication on the use of bone grafting.

Already in 2011, in their milestone paper on open wedge osteotomy, Brosset et al., suggested the safety and efficacy of performing monolateral medial OWHTO with locking plate and early weight bearing protocol without “filling the defect” [20].

Since then, different other authors reported on the safety and efficacy of performing OWHTO without bone grafting. Slevin et al. [8] performed a systematic review of the literature confirming that bone grafting, either autologous, allogenic or synthetic shows no advantages as compared to leaving the gap open, in OWHTO with correction magnitude of less than 10° [8].

Moreover, synthetic augmentation has repeatedly demonstrated to be inferior to other type of augmentation and to no augmentation at all, showing increase risks of nonunion, infection and other complications [7, 8, 22–24], Nevertheless, every paper reporting on SBHTO include a technique with synthetic graft augmentation of the osteotomy gap[13, 10, 14].

In the setting of bilateral OWHTO, very few published research exists. Hernigou et al. [13] performed a safety analysis comparing SBHTO with staged BHTO (35 patients vs 55 patients). They found DVT and length of anticoagulation to be shorter in the SBHTO compared with the sum of both surgeries in the staged setting. The authors concluded that SBHTO is a reasonable treatment option, given that in bilateral disease, staged patients have to face the risks of surgery twice.

Already in 2008, Takeuchi et al. [11] reported good results with a small series of 10 patients undergoing SBHTO with locking plate and Tricalcium-Phosphate synthetic graft, allowing “early” FWB protocol (after 3 weeks). They reported resolution of medial pain and increase in functional scores in all patients, without loss of correction and complications.

Lee et al. [10] compared 22 patients performing SBHTO with 42 patients performing staged BHTO. They found no difference in clinical and functional outcomes at the end of follow up. They reported a significant increase in “loss of correction” in the simultaneous group only at 1 year postoperative, that was not present up to 6 months after surgery, but they did not report nonunion cases. Moreover, they evaluated loss of correction using HKAa (without assessing MPTA or JLCA) that is a whole limb measure and can be affected by intraarticular and femoral confounders. Thus, it is reasonable to think that loss of correction happening after 6 months in a normally healed osteotomy should probably be produced by causes outside the osteotomy stability. In our paper, we evaluated only osteotomy angle differences and found no notable difference in magnitude of correction during follow up.

Neirynck et al. [14] reported a retrospective analysis of 29 patients undergoing SBHTO. They found excellent clinical and functional results at 3, 6 and 12 months for SBHTO. One of the limits of this study, as reported by the author itself, is the absence of “early disability” assessment, with the first evaluation performed 3 months after surgery. In the present paper we also analyze outcomes at 2 months after surgery, finding a significant reduction of the level of activity. This can be due to the caution of the patient in resuming activity after the procedure, and of course could be biased by the indication of surgeon to avoid sports and high impact activity for at least 2 months after surgery. Notably, at the same time, pain scores are significantly reduced, and functional scores are not decreased as compared to baseline, also starting from 2 months after surgery. This information can be useful in helping surgeon counseling patients prior to surgery.

The burden of literature on this topic is quite small, but mostly concord on the good results obtained with a simultaneous bilateral procedure. Yet, all the published papers describe a technique with the use of some bone grafting material, mostly a synthetic B-tricalcium-phosphate wedge. This is the first published series reporting good results and no complication performing SBHTO without any kind of bone grafting and adopting an immediate FWB “at tolerance” protocol. A well performed procedure of cancellous bone mobilization is safe and effective and may act as a “local” bone autograft, as we previously reported [15, 25], leading to bone healing without complications also in bilateral HTO with a magnitude of correction up to 12°.

More studies with a specific design are mandatory to conclude on the superiority of this kind of procedure on SBHTO with bone grafting or even with staged bilateral HTO. In fact, this is not the purpose of this study, that is designed specifically as a preliminary study to assess safety and efficacy of a poorly described procedure.

We are aware that the lack of a control group can be seen as a limitation, but the primary purpose of the present study was to assess the safety and results of SBHTO performed with a yet undescribed procedure. Comparison of results with staged procedure is beyond the scope of this paper.

Important strength points of the present paper are the consistent number of procedures collected, the power of the study and the patients’ evaluation at 2 months postoperative, that provide readers with important information to give the patients during preoperative counseling.

## Conclusion

The main finding of the present study is that in the setting of bilateral symptomatic medial OA in varus, bilateral open wedge high tibial osteotomy can be performed safely and with good results with a simultaneous procedure and without the use of any graft to fill the osteotomy gap. A procedure of metaphyseal cancellous bone mobilization could act as autologous graft to enhance the healing of the osteotomy gap.

## Data Availability

The datasets during and/or analysed during the current study available from the first author on reasonable request.
